# Gastric carcinosarcoma with *FGFR2* amplification under long-term control with pazopanib: a case report and literature review

**DOI:** 10.1186/s12876-022-02432-5

**Published:** 2022-07-28

**Authors:** Hirokatsu Hayashi, Akitaka Makiyama, Naoki Okumura, Itaru Yasufuku, Chiemi Saigo, Tamotsu Takeuchi, Tatsuhiko Miyazaki, Yoshihiro Tanaka, Nobuhisa Matsuhashi, Katsutoshi Murase, Takao Takahashi, Manabu Futamura, Kazuhiro Yoshida

**Affiliations:** 1 Department of Gastroenterological Surgery and Pediatric Surgery, Gifu University Graduate School of Medicine, Gifu, Japan; 2 Cancer Center, Gifu University Hospital, 1-1 Yanagido, Gifu City, 501-1194 Japan; 3 Department of Pathology and Translational Research, Gifu University Graduate School of Medicine, Gifu, Japan; 4 Department of Pathology, Gifu University Hospital, Gifu, Japan; 5 Department of Breast Surgery, Gifu University Graduate School of Medicine, Gifu, Japan

**Keywords:** Gastric carcinosarcoma, Pazopanib, *FGFR2* amplification

## Abstract

**Background:**

Gastric carcinosarcoma is most frequently diagnosed at an advanced stage when the tumor is generally large with invasion into other organs, lymph node metastasis, and distant metastasis. Standard chemotherapy has not been established, and surgery is the only curative treatment. Here, we present a case of postoperative recurrence of gastric carcinosarcoma under long-term tumor control with pazopanib.

**Case presentation:**

A 77-year-old man was referred to our hospital because of nausea and vomiting. Computed tomography and upper gastrointestinal endoscopy revealed a type 1 tumor arising from the gastric antrum and extending into the duodenal bulb. He underwent distal gastrectomy (D2) with Roux-en-Y reconstruction. Histopathologically, the tumor had mixed adenocarcinoma and sarcoma components. According to the tumor–node–metastasis classification, the diagnosis was primary gastric carcinosarcoma pT1bN1M0 stage IB. Liver metastasis was detected 2 months after surgery; multiple lung metastases were detected 17 month after surgery. A genomic profiling test was performed using liver specimens as the patient became refractory to chemotherapy commonly used for gastric cancer, and the test revealed *FGFR2* amplification along with *TP53* R209*, *AKT3* N127D, *NOTCH1* A2036T, and *POLD1* M161I. The patient was treated with pazopanib (800 mg/daily), and the tumor growth was controlled for 11 months.

**Conclusions:**

We report a case of postoperative recurrence of gastric carcinosarcoma under long-term tumor control with pazopanib. This case suggested that pazopanib may be effective in treating gastric carcinosarcoma.

## Background

Gastric carcinosarcoma is most frequently diagnosed at an advanced stage when the tumor is generally large with invasion into other organs, lymph node metastasis, and distant metastasis [1]. Standard chemotherapy has not been established, and surgery is the only curative treatment. Here, we present a case of postoperative recurrence of gastric carcinosarcoma under long-term tumor control with pazopanib.

## Case Presentation

**Fig. 1:**
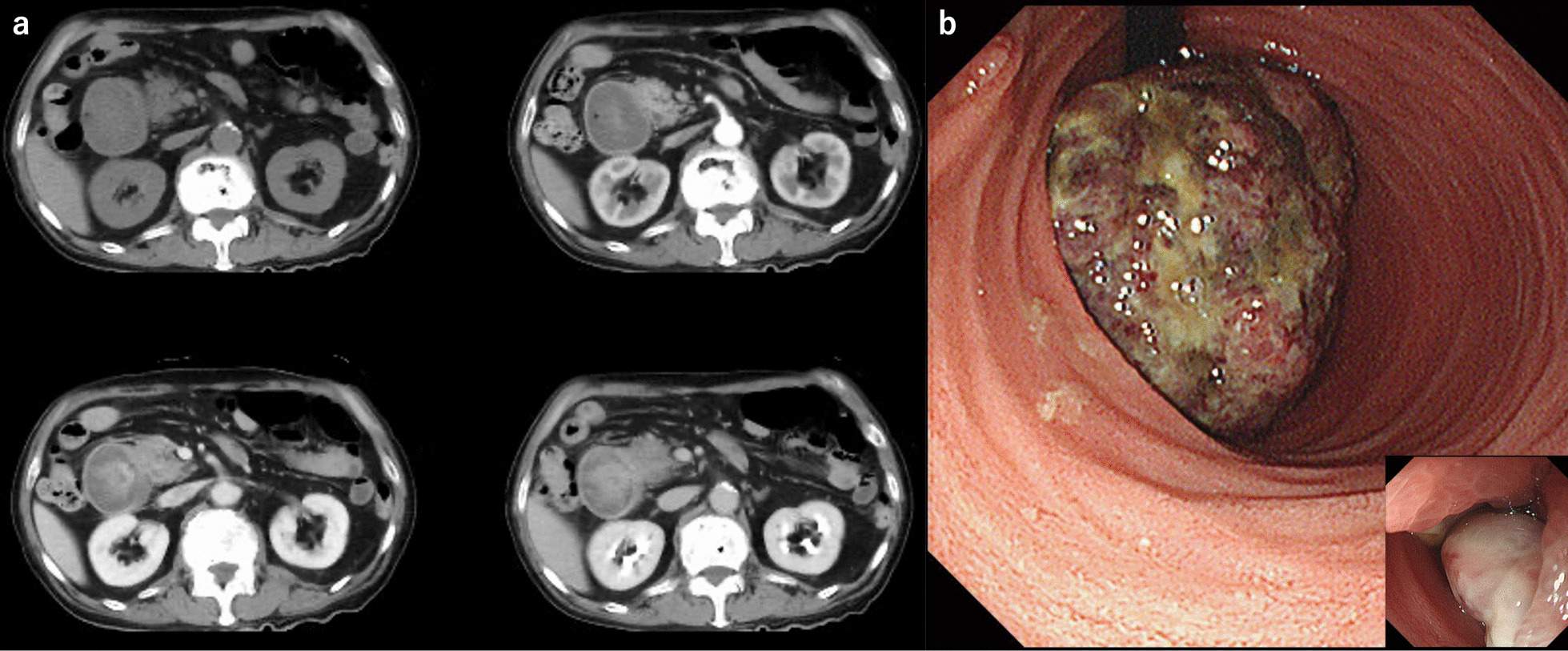
**a** Abdominal contrast computed tomography findings. Gastric outlet obstruction and duodenal wall thickening. **b** Upper gastrointestinal endoscopy findings. A type 1 tumor arising from the gastric antrum and extending into the duodenal bulb

**Fig. 2:**
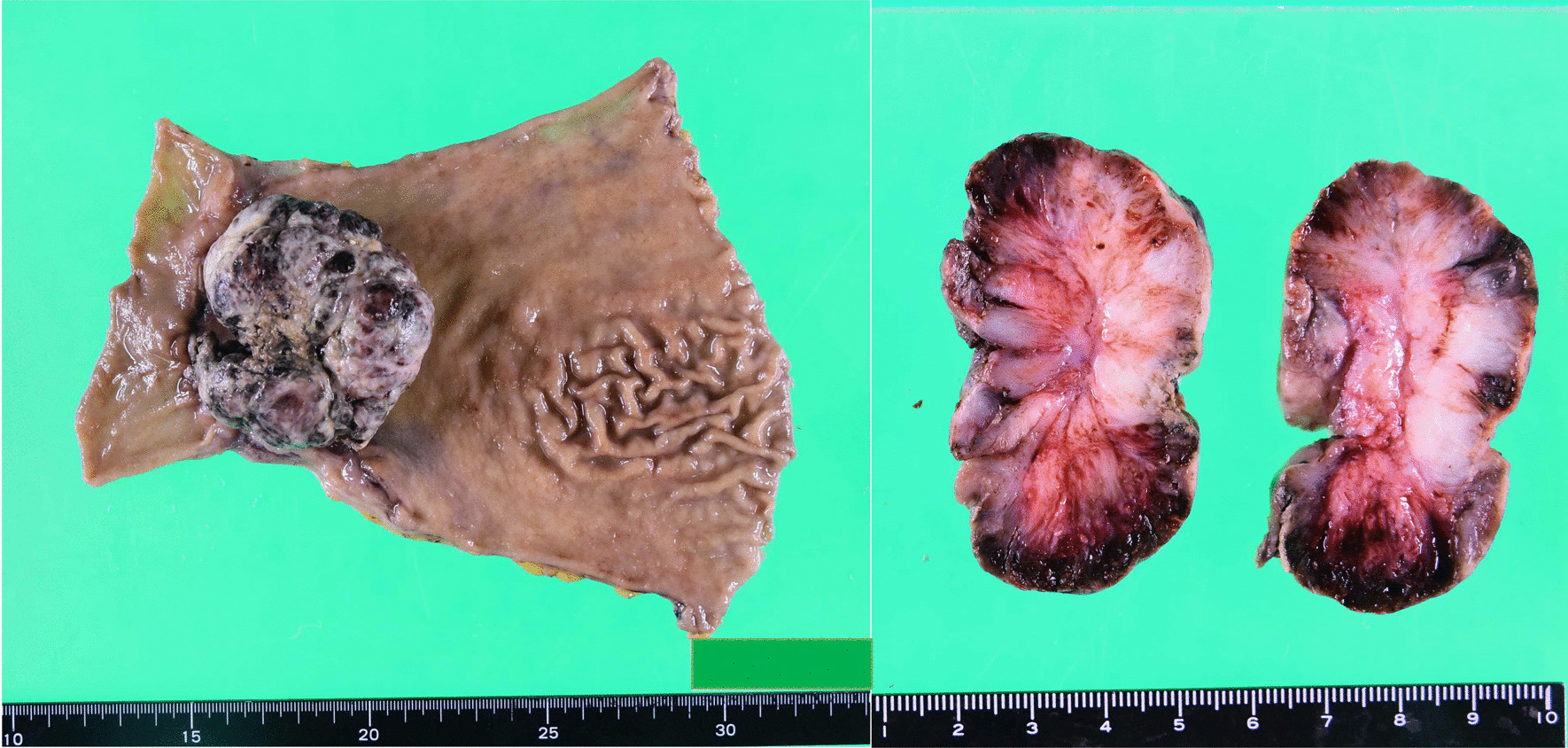
Macroscopic findings. Gastric carcinosarcoma, L, Ant, type 1, 75 × 72 × 35 mm, pT1b(SM), INFb, Ly1a, V1a, pPM0, pDM0, pN1(1/24) (Japanese Classification of Gastric Carcinoma [The 15th Edition])

**Fig. 3:**
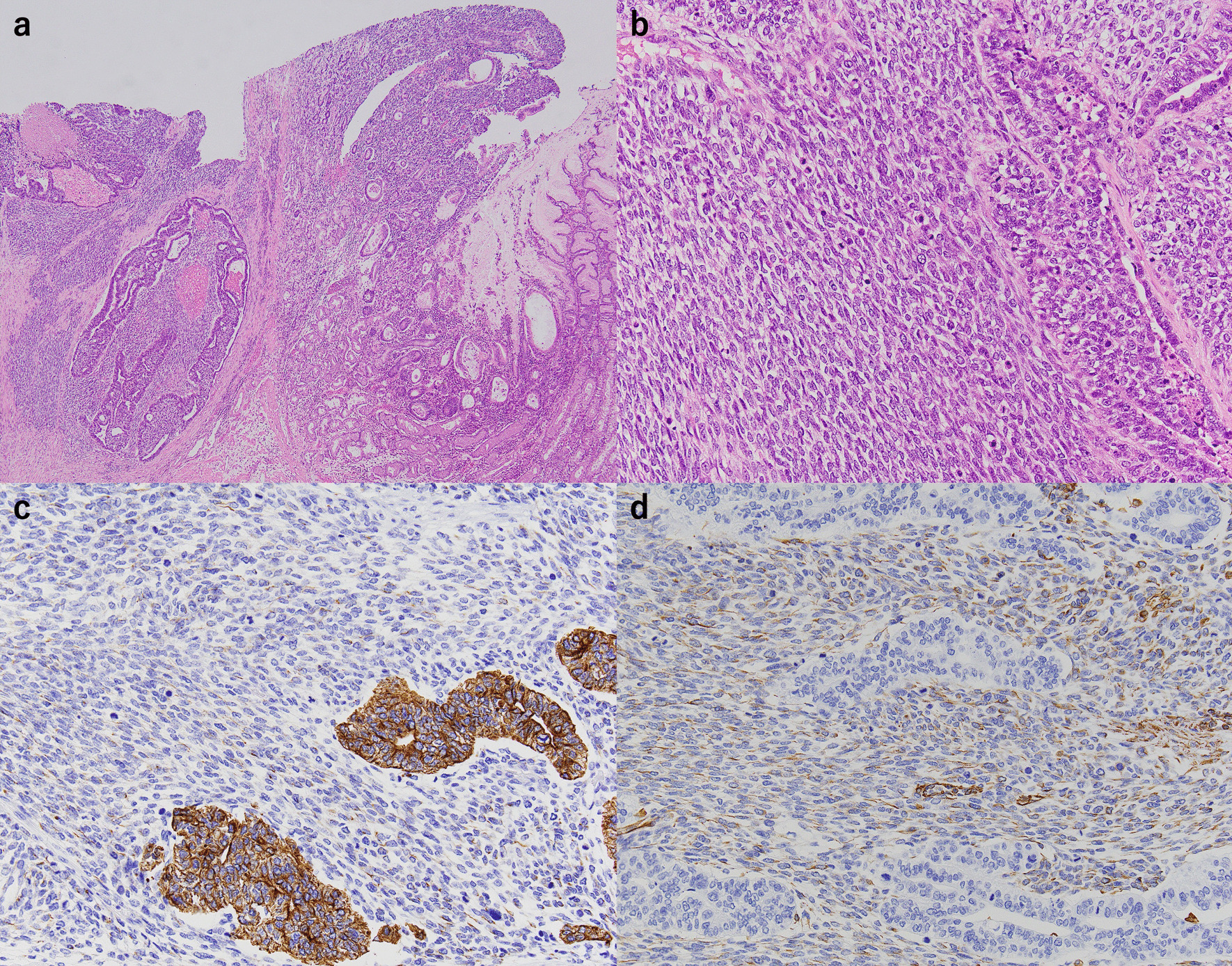
Histopathological findings. **a** The tumor consisted of both adenocarcinoma and sarcoma components. **b** The sarcoma component had features of numerous nuclear mitoses. **c** The carcinoma component was positive for cytokeratin AE1/AE3. **d** The sarcoma component was positive for vimentin

**Fig. 4:**
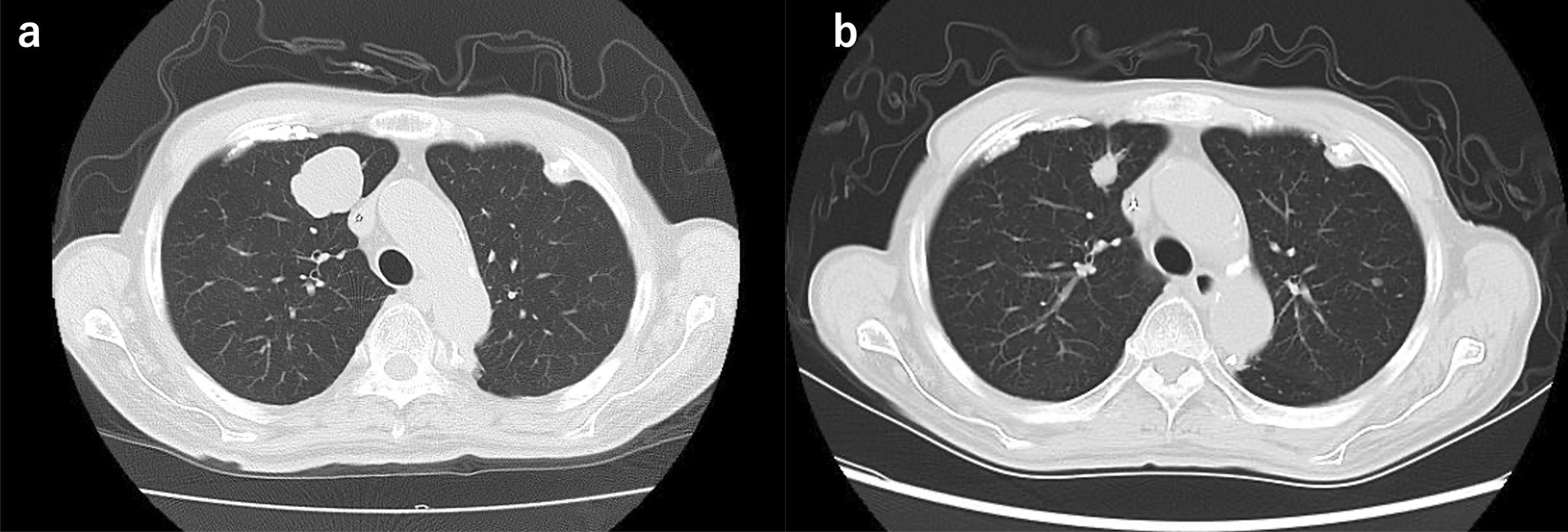
Chest computed tomography findings. Multiple lung metastases in the right upper lobe. **a** Postoperative month 21. **b** Postoperative month 29

A 77-year-old man was referred to our hospital because of nausea and vomiting. He had a past medical history of cerebral infarction, having been prescribed antiplatelet drug, and there was no relevant family history. He has never had any abdominal surgery before. There were no abnormal findings in the physical examination. Computed tomography revealed gastric outlet obstruction and duodenal wall thickening (Fig. 1a). Upper gastrointestinal endoscopy revealed a type 1 tumor arising from the gastric antrum and extending into the duodenal bulb (Fig. 1b). A tissue biopsy specimen showed both adenocarcinoma and spindle sarcomatoid cell components. His serum carcinoembryonic antigen level was 1.3 ng/mL, and his carbohydrate antigen 19–9 level was 7.3 ng/mL. Other laboratory data showed no abnormalities. The patient underwent distal gastrectomy (D2) with Roux-en-Y reconstruction. Histopathologically, the 75 × 72 × 35-mm-sized mass was a type 1 tumor with mixed adenocarcinoma and sarcoma components (Fig. 2). The adenocarcinoma component was composed of a well- to moderately differentiated adenocarcinoma, while the sarcoma component was composed of spindle sarcomatoid cells (Fig. 3a, b). Immunohistochemically, the carcinoma component was positive for cytokeratin AE1/AE3 but negative for vimentin (Fig. 3c). In contrast, the sarcoma component was positive for vimentin but negative for cytokeratin AE1/AE3 (Fig. 3d). Both components were negative for desmin, α-smooth muscle/sarcomeric actin, CD34, CD117 (c-kit), and S100P. Since the tumor was continuous with the existing gastric mucosa without duodenal invasion, primary gastric carcinosarcoma was diagnosed. Although the depth of wall invasion was confined to the submucosa, lymph and blood vessel invasions were noted. Lymph node metastasis was positive in 1 of the 24 dissected lymph nodes and contained the sarcoma component alone. The postoperative diagnosis was pT1bN1M0 stage IB. At postoperative month (POM) 2, liver metastasis was detected with a 22-mm-diameter tumor in Segment 4, and thus, S-1 + cisplatin was initiated. After one cycle of chemotherapy, febrile neutropenia developed (Common Terminology Criteria for Adverse Events v5.0 [CTCAE v5.0] grade 4), and S-1 + cisplatin was discontinued. At POM 5, the liver metastasis enlarged to 43 mm in size, and ramucirumab + paclitaxel was initiated. At POM 10, no increase in liver metastasis was noted, and partial hepatectomy was performed. Histopathological examination of the liver metastasis showed a moderately to poorly differentiated adenocarcinoma without a sarcoma component, and it was classified as grade 1a based on the histological evaluation of the response to chemotherapy. After the second surgery, chemotherapy was discontinued owing to fatigue (CTCAE v5.0 grade 2). At POM 17, multiple lung metastases were detected, and nivolumab was initiated. However, by POM 21, the size of the lung metastases had increased, and nivolumab was discontinued and pazopanib (800 mg/daily) initiated (Fig. 4a). In addition, a genomic profiling test using the OncoGuide™ NCC Oncopanel System was performed using liver specimens, and abnormalities were identified in five cancer-related genes such as fibroblast growth factor receptor (*FGFR*) *2* amplification, *TP53* R209^*^, *AKT3* N127D, *NOTCH1* A2036T, and *POLD1* M161I. During the first 11 months after initiating pazopanib, the lung metastases shrank, with the maximum size decreasing from 34 mm to 17 mm (Fig. 4b). At POM 32, only the lung metastasis in the right upper lobe increased from 17 to 25 mm. At POM 34, brain metastasis was detected with a tumor size of 40 mm, and pazopanib was discontinued. The patient received radiation therapy for brain metastasis and enlarged right upper lobe lung metastasis, which subsequently shrunk. Currently (POM 40), the patient is under treatment-free follow-up.

## Discussion and conclusions

Carcinosarcoma is found in organs such as the uterus, ovaries, bladder, lungs, and esophagus [2]. Gastric carcinosarcoma is an extremely rare disease, with approximately 70 cases reported in the Japanese and English scientific literature [1]. The pathogenesis of gastric carcinosarcoma remains unclear, and standard chemotherapy has not been established [2]. The prognosis is extremely poor, with an estimated mean survival time of 7–10 months [1]. We report a case of postoperative recurrence of gastric carcinosarcoma under long-term tumor control with pazopanib.

Gastric carcinosarcoma is rarely definitively diagnosed preoperatively and is usually detected based on by postoperative pathological examination findings. Gastric carcinosarcoma clinically resembles gastric adenocarcinoma and cannot be easily differentiated either endoscopically or radiologically [1]. Therefore, immunohistochemical analysis of surgical specimens is useful for obtaining a definitive diagnosis of gastric carcinosarcoma [3]. Cytokeratin AE1/AE3, carcinoembryonic antigen, epithelial membrane antigen, chromogranin A, CD56, and synaptophysin are specific markers to identify carcinomatous components, whereas desmin, vimentin, and α-smooth muscle/sarcomeric actin are specific markers to identify sarcomatous components [3]. In this case, cytokeratin AE1/AE3 was expressed in the adenocarcinoma component, and vimentin was expressed in the sarcoma component. Therefore, gastric carcinosarcoma was diagnosed.

No standardized chemotherapy has been established for gastric carcinosarcoma. Some studies have described the effects of chemotherapy, although few studies have shown satisfactory results [4–7]. We selected pazopanib, which has been approved for treating patients with metastatic soft tissue sarcoma. After chemotherapy for gastric cancer was ineffective, the patient was switched to treatment for the sarcoma. Doxorubicin was initially considered; however, because of the patient’s advanced age and decreased wall motion in the cardiac apex, which indicated a decreased myocardial reserve, we decided to use pazopanib, which is generally used after the second-line treatment and is considered to be better tolerated than chemotherapy. Pazopanib is a second-generation small-molecule tyrosine kinase inhibitor with high affinity against vascular endothelial growth factor receptor-1/2/3; and lower affinity against platelet-derived growth factor receptor-α/β, *FGFR-1/2*, and stem cell factor receptor [8]. The PALETTE trial showed a significantly prolonged progression-free survival (4.6 months for patients receiving pazopanib versus 1.6 months for patients receiving placebo) in patients with metastatic soft tissue sarcoma [8]. In this case, the sarcoma component constituted the majority of the tumor and had features of numerous nuclear mitoses that was indicative of high malignancy and strong proliferative potential. This suggests that chemotherapy targeting sarcoma components may be effective and that pazopanib is a suitable treatment.

With the advent of next-generation sequencers, genomic profiling tests for rare cancers have become popular in recent years. In our case, a genomic profiling test with the OncoGuide™ NCC Oncopanel System was performed using liver specimens after the patient became refractory to chemotherapy. Liver specimens selected as recurrent lesions may contain more accurate genetic information of the tumor at the time of pazopanib initiation than the primary lesions do. Abnormalities were identified in five cancer-related genes. Among these, a 23.66-fold amplification of *FGFR2* was identified. Several preclinical models have indicated that abnormalities in *FGFR* may contribute to carcinogenesis [9–11]. *FGFR* amplification often leads to the overexpression of the protein, thereby causing increased accumulation of receptors and activation of downstream signaling pathways. Kim et al. reported that the treatment of gastric cancers with *FGFR2* amplification with pazopanib resulted in a significant decrease in cell survival, while the same treatment showed no growth inhibitory effect on gastric cancers without *FGFR2* amplification [12]. In addition, FGFR is structurally homologous with VEGFR, PDGFR, and other tyrosine kinase receptors, and each receptor has complementary and overlapping functions in promoting angiogenesis. Treatment with a multi-tyrosine kinase inhibitor could target each receptor, potentially leading to synergistic effects [10]. In conclusion, pazopanib, which is the only FGFR inhibitor for the treatment of sarcoma that is covered by insurance in Japan, may be effective against the carcinoma component of gastric carcinosarcoma with *FGFR2* amplification.

Although other genetic abnormalities, such as AKT3 N127D, NOTCH1 A2036T, and POLD1 M161I, may also be involved in carcinogenesis, their significance is unclear. TP53:R209 is considered as a loss-of-function mutation. p53 can activate DNA repair genes or induce apoptosis in the presence of DNA damage. However, treatment for the loss of p53 tumor suppressor function is not common.

Although there is currently no established chemotherapy for gastric carcinosarcoma, pazopanib may be an effective treatment option for carcinosarcoma with highly malignant sarcoma components. Furthermore, consideration of therapeutic agents based on genetic profiling tests is essential for the effective treatment of gastric carcinosarcoma.

## Data Availability

Not applicable.

## Abbreviations

CTCAE: Common terminology criteria for adverse events
*FGFR*: Fibroblast growth factor receptor
POM: Postoperative month
TNM: Tumor–node–metastasis
